# Efficacy of sacubitril‐valsartan and SGLT2 inhibitors in heart failure with reduced ejection fraction: A systematic review and meta‐analysis

**DOI:** 10.1002/clc.24085

**Published:** 2023-07-19

**Authors:** Xingchun Mo, Ping Lu, Xiaojing Yang

**Affiliations:** ^1^ Department of Cardiology, Linping Campus The Second Affiliated Hospital of Zhejiang University School of Medicine Hangzhou China

**Keywords:** all‐cause mortality, cardiovascular mortality, heart failure with reduced ejection fraction, HFrEF, hospitalization for heart failure, meta‐analysis, sacubitril valsartan, SGLT2i, sodium‐glucose cotransporter‐2 inhibitor, SV

## Abstract

**Background:**

Sacubitril‐valsartan (SV) monotherapy has been shown to help patients with Heart failure with reduced ejection fraction (HFrEF), but whether adding a sodium‐glucose cotransporter‐2 inhibitor (SGLT2i) improves treatment results even more is unknown.

**Hypothesis:**

The goal of this study was to look at the efficacy of SV with additional SGLT2i in HFrEF patients.

**Methods:**

For this study, several databases, such as PubMed, EMBASE, Web of Science, and the Cochrane Library, were searched. A coherent search approach was used for data extraction. Review Manager 5.2 and MedCalc were used for conducting the meta‐analysis and bias analysis. A meta‐regression study correlates patient mean age with primary and secondary outcomes.

**Results:**

Seven trials totaling 16 100 patients were included in this meta‐analysis. All‐cause mortality, cardiovascular mortality, and improvement in mean left ventricular ejection fraction (LVEF) were the study's major objectives, while hospitalization for heart failure (HF) was calculated to be its secondary outcome. Our analysis showed that HFrEF patients receiving the combination of SV and SGLT2i had better treatment outcomes than the standard SV monotherapy, with risk ratios of 0.76 (0.65–0.88) for all‐cause mortality, 0.65 (0.49–0.86) for cardiovascular mortality, 1.41 (−0.59 to 3.42) for change in mean LVEF, and 0.80 (0.64–1.01) for hospitalization for HF. According to the regression analysis, older HFrEF patients have higher rates of hospitalization, cardiovascular disease, and overall death.

**Conclusions:**

The combination of SV and SGLT2i may have a greater cardiovascular protective effect and minimize the risk of death or hospitalization due to heart failure in HFrEF.

## Graphical abstract

- Heart failure with reduced ejection fraction (HFrEF) has left ventricular dilatation and unfavorable cardiac remodeling with LVEF < 40%.
- Sacubitril‐valsartan is the first‐line treatment for HFrEF by American College of Cardiology (ACC)/American Heart Association (AHA)/Heart Failure Society of America (HFSA).
- SGLT2 inhibitors also lowers all‐cause and cardiovascular mortality, HF hospitalizations, and significant adverse renal outcomes.
- Present meta‐analysis reported that the combination of SV and SGLT2i may have a greater cardiovascular protective effect and minimize the risk of death or hospitalization due to heart failure in HFrEF

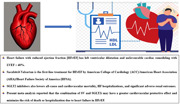

## 1 INTRODUCTION

Heart failure is a medical condition characterized by the inability of the heart muscle to efficiently pump blood. The accumulation of fluid in the lungs can result in dyspnea owing to the pooling of blood in that region.^1^ Certain cardiac conditions can lead to progressive weakening or stiffening of the heart, resulting in impaired ability to adequately fill and eject blood. Congestive heart failure is a medical condition that is marked by reduced cardiac output and pulmonary as well as systemic congestion. It can be attributed to a variety of potential factors.^2^

Heart failure is classified into two distinct categories, namely heart failure with preserved ejection fraction (HFpEF) and Heart failure with reduced ejection fraction (HFrEF), which stand for heart failure with preserved ejection fraction and HFrEF, respectively. This classification is based on the measurement of ejection fraction. Cardiomyocyte loss results in heart failure characterized by reduced ejection fraction, a condition that exhibits a higher prevalence in the male population. In contrast, HFpEF is commonly diagnosed in elderly female patients who have noncardiac comorbidities such as hypertension, type 2 diabetes, and liver disease, among others.^3^, ^4^ Cardiomyopathy and valvular heart disease patients are frequently hospitalized due to heart failure, which is the primary cause of such hospitalizations. This condition is associated with significant mortality and morbidity rates worldwide.^5^

Considerable efforts have been devoted toward identifying the optimal therapeutic approach for heart failure, with the aim of ameliorating symptoms and improving patient prognosis. The clinical manifestations of heart failure that result in congestion are commonly managed through the administration of diuretics and digitalis. Diuretics are utilized to reduce the workload on the heart, while digitalis is employed to enhance the contractile function of the myocardium.^6^ The utilization of angiotensin receptor inhibitors, beta‐blockers, angiotensin‐converting enzyme inhibitors (ACEI), and mineralocorticoid receptor antagonists has been prompted by their observed efficacy in enhancing symptom and prognosis outcomes. The efficacy of these medications has been demonstrated in enhancing the quality of life, ventricular remodeling, reducing mortality, and hospitalization rates, as evidenced by studies.^7^, ^8^

Recently, it has been observed that two new drugs, namely sodium‐glucose cotransporter‐2 inhibitor (SGLT2i) and angiotensin receptor neprilysin inhibitor (ARNI), exhibit enhanced cardiovascular protection in patients with heart failure as compared to conventional therapy.^9^ The combination of valsartan and sacubitril, known as ARNI, has demonstrated efficacy in enhancing hemodynamic and neurohormonal outcomes among individuals with heart failure. According to a study, it was observed that the efficacy of the drug in reducing the risk of cardiovascular death or hospitalization for patients with HFrEF was higher than that of ACEI.^10^

The SGLT2i is a new hypoglycemic drug that has demonstrated various pleiotropic effects in patients diagnosed with diabetes mellitus (DM). These effects include the reduction of blood pressure, decreased cardiovascular risk, and preservation of renal function.^11^, ^12^ The concurrent administration of these medications was previously considered the optimal therapeutic approach for individuals diagnosed with heart failure characterized by diminished ejection fraction. Two recent studies, namely Huang et al.^13^ and Cordovez et al.^14^ conducted systematic reviews and meta‐analyzes on the effects of SGLT2i on individuals with a history of heart failure. The findings of both studies indicate that the use of SGLT2i is associated with a significant reduction in the risk of cardiovascular death and a decreased need for hospitalization due to heart failure.

Although these studies showed that SGLT2i could help patients with HFrEF, it is still unclear whether or not using both drugs together would be more effective than using either one alone. As a result, we conducted this systematic review and meta‐analysis to delve into these issues more deeply with the available research evidence.

### 1.1 Objective

The objective of this study was to investigate the efficacy of SV with added SGLT2i in patients with HFrEF.

## 2 MATERIALS AND METHODS

The present study adhered to the PRISMA normative recommendations^15^ and was registered under the number ZUSM#IRB‐1040/2022.

### 2.1 Search strategy

This meta‐analysis is predicated on a comprehensive search that was conducted in the databases of Medline (via PubMed), Cinahl (via Ebsco), Scopus, and WoS from the year 2000 through 2023. An inclusive literature search was conducted with the following inclusion criteria: (i) clinical randomized clinical trials or retrospective studies related to the use of SV and SGLT2i for HFrEF patients; (ii) patients with HFrEF with left ventricular ejection fraction (EF) < 40%; and (iii) Studies providing primary outcome data: All‐cause mortality, cardiovascular mortality or hospitalization for heart failure and (iv) articles published in English language using the following keywords: (I) “Sodium‐glucose cotransporter‐2 inhibitor” OR SGLT2i; (II) “Sacubitril Valsartan” OR SV; (III) “Heart failure with reduced ejection fraction” OR HFrEF; (IV) “Dapagliflozin”; (V) “Empagliflozin”; (VI) “All‐cause mortality”; (VII) “Cardiovascular mortality”; (VIII) “Mean Left ventricular ejection fraction” OR LVEF; (IX) “Hospitalization for heart failure”; (X) “Meta‐analysis.”

We used the Boolean operator “AND” to join the Medical Subject Headings with the text keywords within the search strategy. The search results were initially screened for duplicates, and then the remaining articles were screened based on their titles and abstracts. All eligible studies' full texts were then retrieved, assessed for inclusion and exclusion using the predetermined inclusion and exclusion criteria, and added in accordance with PRISMA standards.

### 2.2 Inclusion and exclusion criteria

Included were reports of research comparing the effectiveness of SV plus SGLT2i versus SV monotherapy in the treatment of HFrEF. The studies chosen span the years 2019 and 2023. Abstracts, studies without enough data for a 2 × 2 table, and related research that did not meet the inclusion criteria were all disqualified from inclusion in the present study. Two writers (X. M. and P. L.) independently searched the appropriate databases for relevant studies, while X. Y. researcher worked separately to extract the characteristics of the included studies^16^, ^17^, ^18^, ^19^, ^20^, ^21^, ^22^ and event data with key variables. Through discussions, differences of opinion were reconciled.

### 2.3 Evaluation of the analytical standard and source of heterogeneity

The methodological validity of the studies included in the analysis was assessed by two reviewers (X. M. and P. L.), who also calculated the heterogeneity of the included studies. The author (X. Y.) assumed the responsibility of resolving any form of disagreement that may have arisen among fellow authors (X. M. and P. L.). The study utilized Cochran statistics and the *I* ^2^ index to examine heterogeneity in a random bivariate mode. The analysis was conducted using RevMan (Review Manager) software, version 5.3, developed by The Nordic Cochrane Center in Copenhagen, Denmark.^23^ The sources of heterogeneity that were examined include the utilization of randomized controlled trials versus retrospective studies, variations in the number of patients with HFrEF, differences in study designs, and distinct data analysis techniques.

### 2.4 Evaluation of risk of bias

The RevMan software was utilized to assess the caliber of the studies incorporated in this meta‐analysis, and subsequently, a risk of bias graph and summary were generated. This table documents information pertaining to the generation of random sequences, allocation concealment, participant and staff blinding, outcome assessment blinding, inadequate outcome data, selective reporting, and other potential sources of bias. By utilizing the table, we were able to allocate a rating of “low,” “high,” or “some concern” to each factor pertaining to the quality of the study. The investigation was conducted by two distinct researchers, namely X. M. and P. L., who worked independently. In the event of unresolved disagreements, a third researcher, X. Y., intervened. Publication bias was assessed using the MedCalc software^24^ through the application of Begg's test^25^ and Deek's funnel plot.^26^

### 2.5 Statistical analysis

A meta‐analysis was conducted using the RevMan and MedCalc software. Statistical analysis was conducted using risk ratios (RR) and mean differences calculated from the event data, which were organized into a 2 × 2 table. Forest plots were created to visualize the results.^27^, ^28^, ^29^, ^30^ The heterogeneity of the studies^31^ was evaluated based on the *χ* ^2^ value, τ^2^ value, *df* value, *I* ^2^ value, *z* value, and *p* value. When the level of heterogeneity was 50% or higher, a random‐effects model was employed for the purpose of data analysis. Conversely, in cases where the level of heterogeneity was at or below 50%, a fixed‐effects model was employed. The present investigation reported categorical results in the form of odds ratios (OR), RR, and 95% confidence intervals (CI), while continuous variables were expressed as mean differences (MD) accompanied by their corresponding 95% CIs. The present meta‐analysis employed a significance threshold of 0.05 for the *p* value. Subgroup analysis was conducted based on the study design. A meta‐regression analysis was carried out to investigate the degree of association that existed between the mean age of the patients and the primary and secondary outcomes.

## 3 RESULTS

### 3.1 Literature search results

By conducting electronic searches across multiple databases, a sum of 350 studies was identified. A total of 147 records were eliminated on account of duplication, while 203 records underwent evaluation. In addition, a total of 172 studies were excluded after conducting a thorough screening of their titles and abstracts, resulting in a final pool of 31 studies for further evaluation. Fifteen out of the total of 24 studies were excluded from the analysis due to their failure to report the intended outcome, while nine studies were deemed ineligible for inclusion based on the established criteria. This meta‐analysis selected a total of seven papers published between 2019 and 2022 that met the inclusion criteria, which involved the utilization of SV and SGLT2i for patients with HFrEF. The details of the selected papers are presented in Supporting Information: Figure 1.

Table 1 presents the salient features of the studies incorporated in this meta‐analysis, encompassing a cumulative cohort of 16 100 patients with HFrEF. The aforementioned text provides information regarding the author of the study, the year of publication, the age range of the participants, the journal of publication, the study design, the total sample size, the number of participants in both the experimental and control groups, and the principal findings of the meta‐analysis.

**Table 1:** Characteristics of the included studies.

References	Country	Journal of publication	Age (EG vs. CG) Mean ± SD	Sample size	Sample size EG/CG	Type of study	Primary and secondary outcomes	Follow up
Hsiao et al.^16^	Taiwan	*International Journal of Cardiology*	61.9 ± 13.1 vs. 68.8 ± 12.7	2312	169/338	Retrospective cohort study	All‐cause mortality, Hospitalization for HF	12 months
Jiang et al.^17^	China	*Frontiers in Cardiovascular Medicine*	67.6 ± 12.6 vs. 70.3 ± 12.9	136	72/64	Retrospective Study	Change in LVEF	6 months
Karabulut et al.^18^	Turkey	*Angiology*	67.3 ± 9.7 vs. 65.2 ± 10.23	244	81/163	Retrospective Study	Cardiac and all‐cause mortality, hospitalization for HF	30 months
Larsen et al.^19^	Denmark	*American Heart Journal Plus Cardiology Research and Practice*	68 ± 10 vs. 63 ± 10	190	58/132	The EMPIRE‐HF trial	Change in LVEF	3 months
Murray et al.^20^	United States of America	*The New England Journal of Medicine*	66.2 ± 11.0 vs. 66.5 ± 10.8	4744	2373/2371	The DAPA‐HF trial	Mortality from cardiovascular causes, hospitalization for HF	12 months
Packer et al.^21^	United States of America	*European Heart Journal*	66.5 ± 11.2 vs. 66.5 ± 11.4	3730	727/3003	The EMPEROR‐Reduced trial	All‐cause mortality, Hospitalization for HF	12 months
Solomon et al.^22^	United States of America	*JACC (Journal of the American College of Cardiology): Heart Failure*	66.2 ± 11.0 vs. 66.4 ± 10.9	4744	508/4236	The DAPA‐HF Trial	All‐cause mortality, hospitalization for HF	8 months

Abbreviations: CG, control group; DAPA, DAPA‐HF (dapagliflozin and prevention of adverse outcomes in heart failure); EG, experimental group; HF, heart failure; LVEF, left ventricular ejection fraction.

### 3.2 Meta‐analysis results

The meta‐analysis was conducted using the RevMan and MedCalc software applications. The findings are subsequently examined in the following manner:

#### 3.2.1 Risk of bias assessment

The evaluation of potential bias was carried out utilizing a pre‐established survey instrument, and the findings are exhibited in Supporting Information: Table 1.

**Figure 1:**
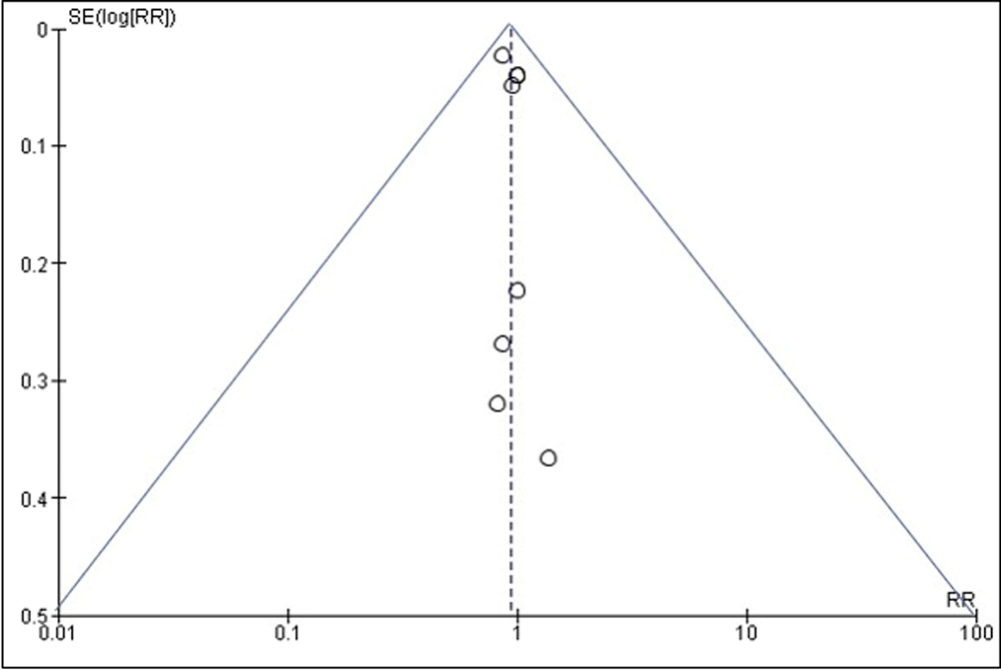
Funnel plot for publication bias.

Supporting Information: Figure 2 provides a summary of the potential for bias, whereas Supporting Information: Figure 3 presents a graphical representation of the potential for bias. Among the seven studies that were included in the analysis, it was found that three of them exhibited a low risk of bias. Meanwhile, three studies were classified as having a moderate risk of bias, primarily due to confounding and missing data. Last, one study was identified as having a high risk of bias, which was attributed to the measurement of exposure. The present meta‐analysis exhibits a minimal likelihood of publication bias, as indicated by the graphical representation in Figure 1 and the lack of statistical significance in the *p* values of Begg's test (*p* > .05).^32^

#### 3.2.2 Statistical assessment

**Figure 2:**
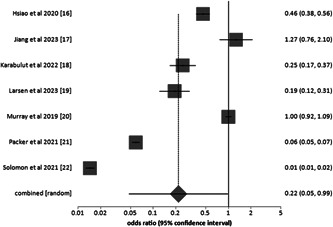
Forest plot for overall risk ratio.

Statistical assessment was performed using the event data extracted from the included studies and the results are discussed in Supporting Information: Table 2. We obtained the overall pooled OR of 0.22 (95% CI 0.05–0.99) with heterogeneity of *χ* ^2^ value 3.59, *df* value 1, *I* ^2^ value 99%, and *p* < .001. The respective forest plot is shown in Figure 2. Since the *I* ^2^ value is >50%, a random effect model was used.

The primary and secondary outcomes of the included studies were also assessed separately and the results are discussed as below:

##### Results for primary outcome: (Figure 3)

**Figure 3:**
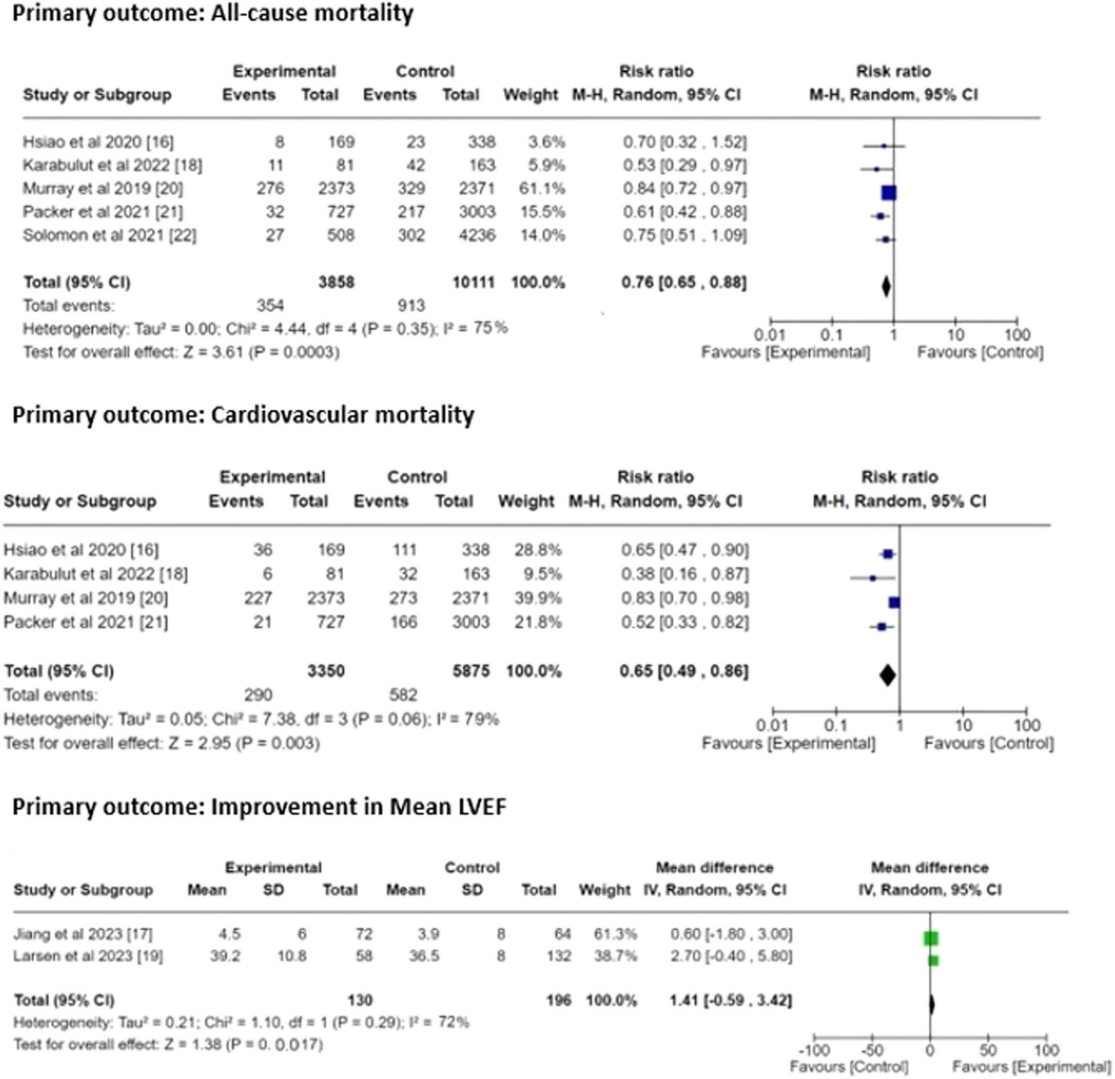
Forest plot risk ratio for primary outcomes.

(i) **All‐cause mortality**:
  We obtained the pooled RR = 0.76 (95% CI 0.65–0.88) with the heterogeneity of τ^2^ value 0.00, *χ* ^2^ value 4.44, *df* value 4, *I* ^2^ value 75%, *z* value 3.61, and *p* = .0003.
(ii) **Cardiovascular mortality**:
  We obtained the pooled RR = 0.65 (95% CI 0.49–0.86) with the heterogeneity of τ^2^ value 0.05, *χ* ^2^ value 7.38, *df* value 3, *I* ^2^ value 79%, *z* value 2.95, and *p* = .003.
(iii) **Change in mean LVEF**:
  We obtained the MD = 1.41 (95% CI 0.59–3.42) with the heterogeneity of τ^2^ value 0.21, *χ* ^2^ value 1.10, *df* value 1, *I* ^2^ value 72%, *z* value 1.38, and *p* = .0017.

##### Results for secondary outcome

**Figure 4:**
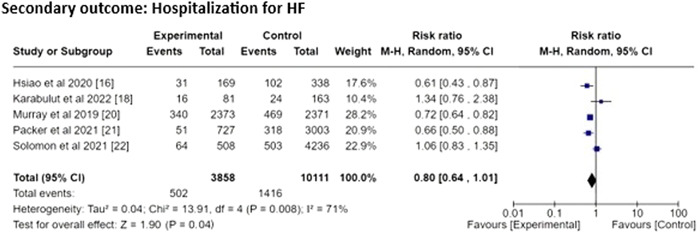
Forest plot risk ratio for secondary outcome.

(i) **Hospitalization for HF**:

We obtained the pooled RR = 0.80 (95% CI 0.64–1.01) with the heterogeneity of τ^2^ value 0.04, *χ* ^2^ value 13.91, *df* value 4, *I* ^2^ value 71%, *z* value 1.90, and *p* = .04. (Figure 4).

The RR and OR values <1 indicate a high likelihood of achieving better outcomes with the combination therapy of SV and SGLT2i for HFrEF patients as compared to standard SV monotherapy, as it reduces cases of all‐cause mortality, cardiovascular mortality, and hospitalizations for heart failure significantly and improves the mean LVEF value to a substantial degree.

The findings of regression analysis indicate that elderly patients with HFrEF are susceptible to increased rates of hospitalization, a changed mean LVEF, as well as cardiovascular and all‐cause mortality as shown in Supporting Information: Figures 4 and 5.

## 4 DISCUSSION

HFrEF is distinguished by the enlargement of the left ventricle and unfavorable cardiac remodeling, resulting in an LVEF of 40% or less.^33^ The present meta‐analysis aimed to evaluate the effectiveness of SV monotherapy compared to the combination of SV and Sodium‐glucose cotransporter 2 inhibitors (SGLT2i) in the management of HFrEF. The medication SV is a composite of the neprilysin inhibitor sacubitril and valsartan. This compound functions as an angiotensin receptor II blocker and neprilysin inhibitor, thereby inhibiting both the natriuretic peptide system and the renin–angiotensin–aldosterone system. The ARNI medication has been observed to enhance vasodilation and diuresis by reducing nitric oxide synthase, while concurrently mitigating vasoconstriction and salt retention.

The dual mode of action has led to enhanced clinical outcomes among individuals diagnosed with HFrEF.^34^, ^35^ The American College of Cardiology, American Heart Association, and Heart Failure Society of America have issued guidelines that advocate for the use of ARNI as the primary treatment for HFrEF. This recommendation is based on their research and analysis. SGLT2i are of comparable significance as a favorable therapeutic intervention for patients with HFrEF. Empagliflozin or dapagliflozin SGLT2 inhibition is recommended as an adjunct to conventional medical care for HFrEF, owing to its significant advantages in decreasing all‐cause and cardiovascular mortality, HF hospitalizations, and severe adverse renal outcomes.^36^, ^37^, ^38^

The present meta‐analysis aimed to assess the advantages of the combination therapy of SGLT2i and SV in the management of HFrEF. To achieve this, we included four recently conducted clinical trials, namely the EMPIRE‐HF trial, the DAPA‐HF trials, and the EMPEROR‐Reduced trial, along with three retrospective studies. The retrospective studies conducted by Hsiao et al.,^16^ Jiang et al.,^17^ and Karabulut et al.^18^ examined the impact of the combination of SGLT2i and ARNI on patients with heart failure and reduced ejection fraction and DM. The studies concluded that this combination therapy was well‐tolerated among diabetic patients with HFrEF and was linked to a reduced risk of hospitalization due to heart failure.

The efficacy of combining SV with SGLT2i was assessed in several clinical trials, including Larsen et al.'s EMPIRE‐HF trial, Murray et al.'s DAPA‐HF trial, Packer et al.'s EMPEROR‐Reduced trial, and Solomon et al.'s DAPA‐HF trial. The results indicated that the combined treatment of SV and SGLT2i produced significant advantages. The meta‐analysis of the studies included in this research indicates that the combination of SV and SGLT2i is superior in reducing all‐cause mortality, cardiac death outcomes, and hospitalization incidence. The results show a significant decrease in all‐cause mortality with an RR = 0.76 (0.65–0.88), cardiovascular mortality with RR = 0.65 (0.49–0.86), and hospitalization for HF with an RR = 0.80 (0.64–1.01).

In addition, a significant alteration in the average LVEF was observed through the implementation of the combined therapy, resulting in an MD of 1.41 (−0.59 to 3.42). All of the aforementioned findings exhibited statistical significance, as indicated by an overall effect *p* value below .05. The results obtained are in line with the research conducted by Yan et al. in 2021,^39^ which involved the systematic examination and statistical analysis of six clinical trials. The researchers in these studies posited that the concomitant administration of SGLT2i and ARNI results in an enhanced cardiovascular safeguarding effect, as evidenced by a hazard ratio of HR 0.68 and a 95% CI spanning from 0.53 to 0.89. Lee et al.^40^ conducted a meta‐analysis and systematic review, which revealed that the co‐administration of SGLT2i and ARNI resulted in a significant improvement in LVEF and LV remodeling. A comprehensive review and meta‐analysis by Teo et al.^41^ found that sacubitril and valsartan cure heart failure better than SGLT2is. SV improved heart failure hospitalization, cardiovascular mortality, and long‐term blood pressure. Banerjee et al.^42^ conducted a thorough review and meta‐analysis, which revealed that SGLT2i served as a fundamental therapeutic intervention for patients diagnosed with HF with preserved and mildly reduced ejection fraction, irrespective of their diabetic status.

The results of this meta‐analysis offer a novel therapeutic approach that could potentially enhance the prognosis of individuals with HFrEF. However, it is crucial to acknowledge that this investigation has several constraints that warrant careful attention.

## 5 LIMITATIONS

Within the scope of this analysis, there are some limitations that need to be considered. To begin, rather than using the data from each individual participant, we used aggregated data from the study. Second, the criteria for inclusion of HFrEF patients and the subsequent follow‐up length in various trials were slightly varied from one another, which may have contributed to some degree of internal heterogeneity. Third, the specific description of primary outcomes differed somewhat but noticeably between the many studies that were considered. Last but not least, this particular search only returned results for publications written in English, which may have introduced some bias into the paper selection process.

## 6 CONCLUSIONS

The administration of SGLT2is has been shown to reduce the likelihood of cardiovascular mortality or hospitalization due to heart failure in individuals with HFrEF, regardless of their diabetic status. The aforementioned decrease is both substantial and secure. Both SGLT2i and SV are linked with outcomes that are equivalent to the prevention of cardiovascular mortality or hospitalization due to heart failure. Furthermore, the utilization of SGLT2i in combination with SV therapy results in an enhanced cardiovascular protective effect.

## AUTHOR CONTRIBUTIONS

**Xingchun Mo**: Concept and designed the study. **Ping Lu** and **Xiaojing Yang**: Analyzed data and drafting of the manuscript. **Xingchun Mo** and **Ping Lu**: Collected the data and helped in data analysis. **Xiaojing Yang**: Proofreading and final editing along with guarantor of the manuscript. All authors read and approved the final version of the manuscript.

## CONFLICT OF INTEREST STATEMENT

The authors declare no conflict of interest.

## Supporting information

Supporting information.

Supporting information.

Supporting information.

Supporting information.

Supporting information.

Supporting information.

Supporting information.

## Data Availability

All data generated or analyzed during this study are included in this article. Further enquiries can be directed to the corresponding author.
