# Supplementary figures and images for: Efficacy of sacubitril‐valsartan and SGLT2 inhibitors in heart failure with reduced ejection fraction: A systematic review and meta‐analysis
Source: Clin Cardiol. 2023 Jul 19;46(10):1137–45. doi: 10.1002/clc.24085 (PMC10577570; doi:10.1002/clc.24085)

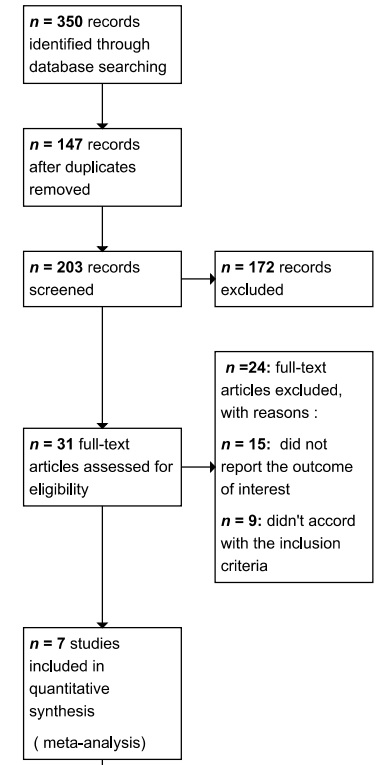

Supplement: Supplementary file 1 — Supporting information. [file CLC-46-1137-s001.jpg]

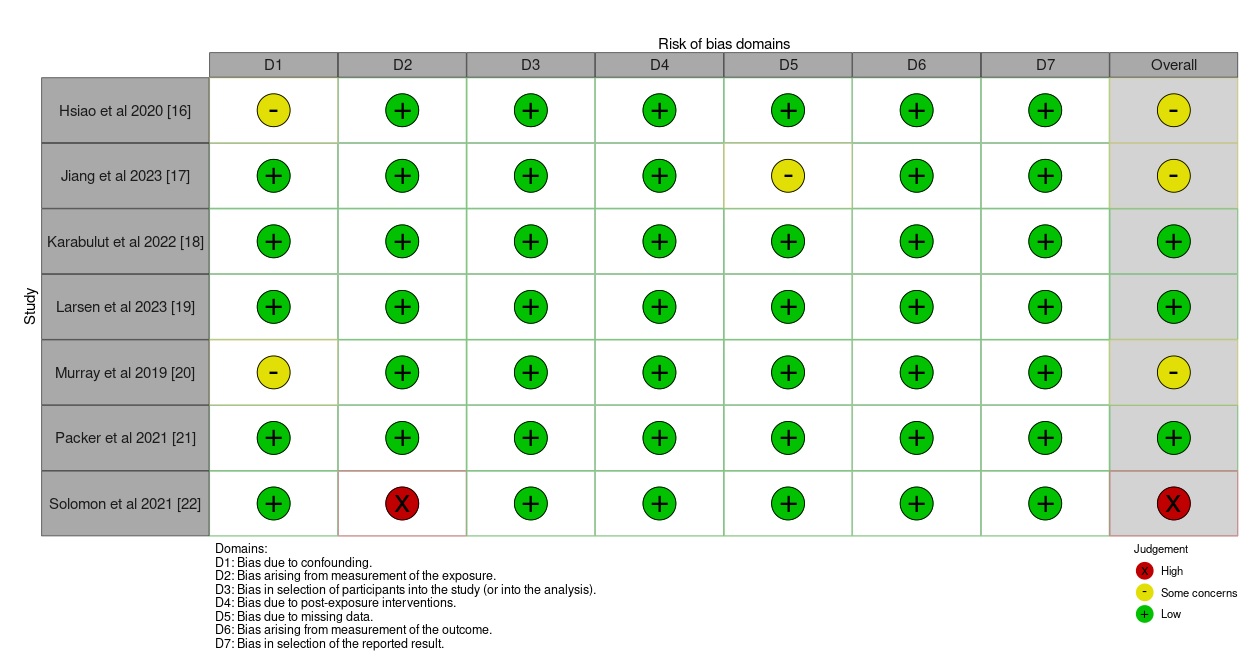

Supplement: Supplementary file 2 — Supporting information. [file CLC-46-1137-s006.jpeg]

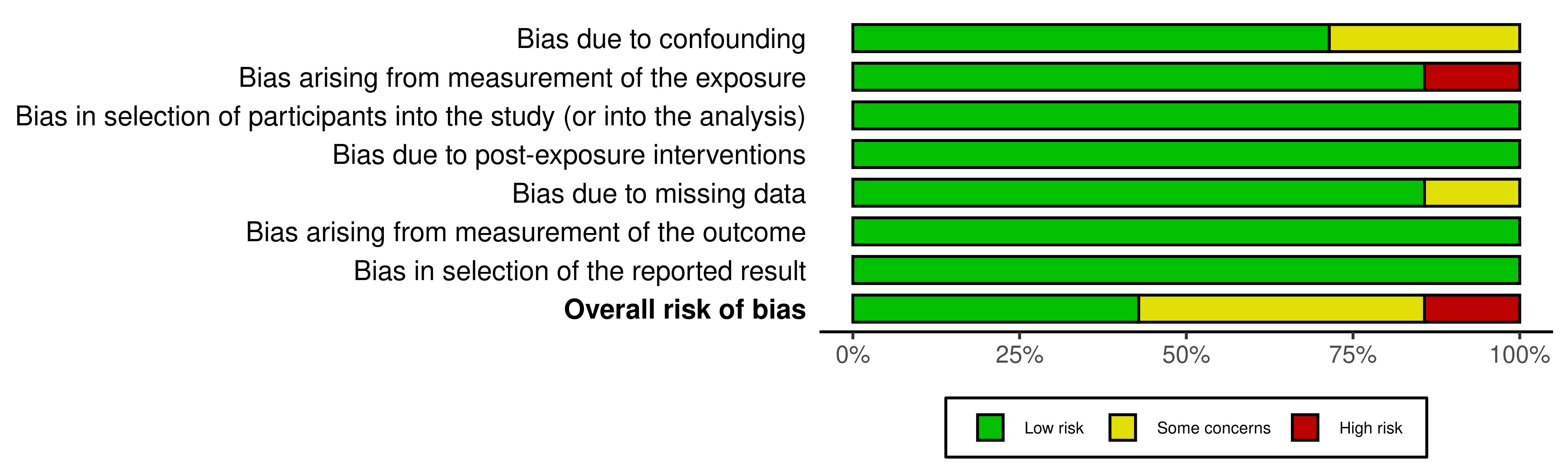

Supplement: Supplementary file 3 — Supporting information. [file CLC-46-1137-s004.jpeg]

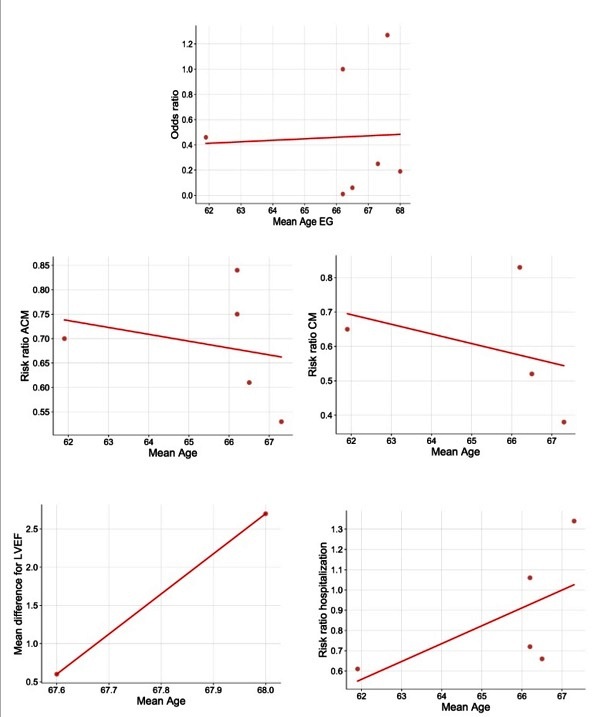

Supplement: Supplementary file 4 — Supporting information. [file CLC-46-1137-s003.jpg]

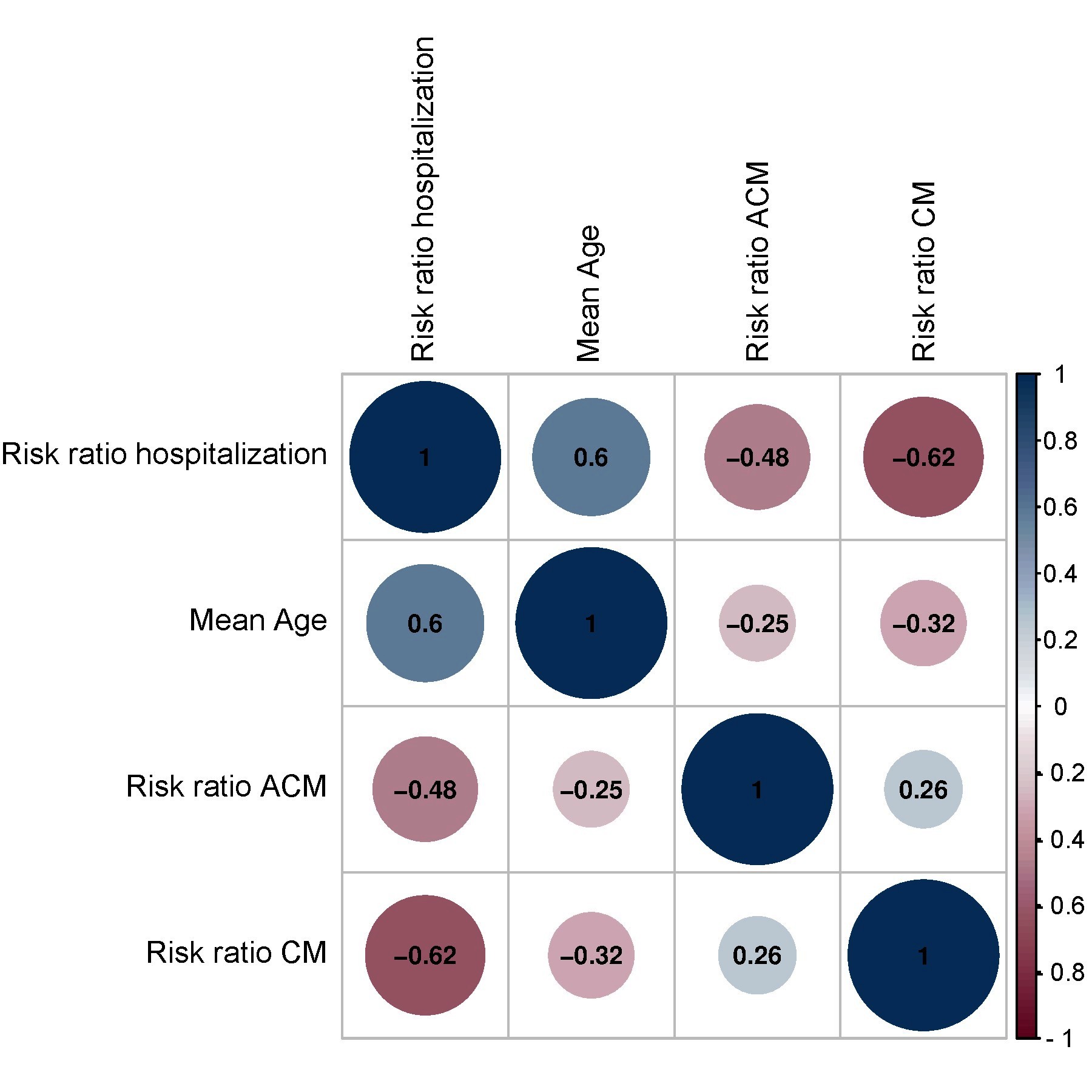

Supplement: Supplementary file 5 — Supporting information. [file CLC-46-1137-s007.jpeg]
