# Supplementary material for: Efficacy of sacubitril‐valsartan and SGLT2 inhibitors in heart failure with reduced ejection fraction: A systematic review and meta‐analysis
Source: Clin Cardiol. 2023 Jul 19;46(10):1137–45. doi: 10.1002/clc.24085 (PMC10577570; doi:10.1002/clc.24085)
Supplement: Supplementary file 6 — Supporting information. [file CLC-46-1137-s005.docx]

**Supplementary Table 1: Risk Assessment of Included Studies**

| **Study ID and Year** | **Hsiao et al 2020 [16]** | **Jiang et al 2023 [17]** | **Karabulut et al 2022 [18]** | **Larsen et al 2023 [19]** | **Murray et al 2019 [20]** | **Packer et al 2021 [21]** | **Solomon et al 2021 [22]** |  |
| --- | --- | --- | --- | --- | --- | --- | --- | --- |
| Did the study avoid inappropriate exclusions | Y | Y | Y | Y | Y | Y | Y |  |
| Did all patients receive the same reference standard | Y | Y | Y | Y | Y | Y | Y |  |
| Were all patients included in the analysis | N | N | N | N | N | N | N |  |
| Was the sample frame appropriate to address the target population? | Y | Y | Y | Y | Y | Y | Y |  |
| Were study participants sampled in an appropriate way? | Y | Y | Y | Y | Y | Y | Y |  |
| Were the study subjects and the setting described in detail? | Y | Y | Y | Y | Y | Y | Y |  |
| Were valid methods used for the identification of the condition? | Y | Y | Y | Y | Y | Y | Y |  |
| Was the condition measured in a standard, reliable way for all participants? | Y | Y | Y | Y | Y | Y | Y |  |
