# Supplementary material for: Efficacy of sacubitril‐valsartan and SGLT2 inhibitors in heart failure with reduced ejection fraction: A systematic review and meta‐analysis
Source: Clin Cardiol. 2023 Jul 19;46(10):1137–45. doi: 10.1002/clc.24085 (PMC10577570; doi:10.1002/clc.24085)
Supplement: Supplementary file 7 — Supporting information. [file CLC-46-1137-s002.docx]

| **Study Outcome** | **Effect Estimated** | **p value** |
| --- | --- | --- |
| **Overall effect** | **Pooled OR:** .22 [0.05-0.99] | < 0.001. |
| **Primary Outcomes:** | | |
| All-cause mortality | Pooled RR: 0.76 [ 0.65 – 0.88] | 0.0003 |
| Cardiovascular mortality | Pooled RR: 0.65 [ 0.49 – 0.86] | 0.003 |
| Change in LVEF | Pooled Mean difference: 1.41 [-0.59 – 3.42] | 0.017 |
| **Secondary outcome:** | | |
| Hospitalization for HF | Pooled RR: 0.80 [0.64 -1.01] | 0.04 |

**Supplementary Table 2: Statistical Summary of the StudyOutcomes**
